# Principles of Genetic Engineering

**DOI:** 10.3390/genes11030291

**Published:** 2020-03-10

**Authors:** Thomas M. Lanigan, Huira C. Kopera, Thomas L. Saunders

**Affiliations:** 1 Biomedical Research Core Facilities, Vector Core, University of Michigan, Ann Arbor, MI 48109, USA; lanigant@umich.edu (T.M.L.); chongh@umich.edu (H.C.K.); 2 Department of Internal Medicine, Division of Rheumatology, University of Michigan, Ann Arbor, MI 48109, USA; 3 Department of Human Genetics, University of Michigan, Ann Arbor, MI 48109, USA; 4 Biomedical Research Core Facilities, Transgenic Animal Model Core, University of Michigan, Ann Arbor, MI 48109, USA; 5 Department of Internal Medicine, Division of Genetic Medicine, University of Michigan, Ann Arbor, MI 48109, USA

**Keywords:** CRISPR/Cas9, embryonic stem (ES) cells, genetic engineering, gene targeting, homologous recombination, microinjection, retroviruses, transgenic mice, transgenic rats, transposons, vectors

## Abstract

Genetic engineering is the use of molecular biology technology to modify DNA sequence(s) in genomes, using a variety of approaches. For example, homologous recombination can be used to target specific sequences in mouse embryonic stem (ES) cell genomes or other cultured cells, but it is cumbersome, poorly efficient, and relies on drug positive/negative selection in cell culture for success. Other routinely applied methods include random integration of DNA after direct transfection (microinjection), transposon-mediated DNA insertion, or DNA insertion mediated by viral vectors for the production of transgenic mice and rats. Random integration of DNA occurs more frequently than homologous recombination, but has numerous drawbacks, despite its efficiency. The most elegant and effective method is technology based on guided endonucleases, because these can target specific DNA sequences. Since the advent of clustered regularly interspaced short palindromic repeats or CRISPR/Cas9 technology, endonuclease-mediated gene targeting has become the most widely applied method to engineer genomes, supplanting the use of zinc finger nucleases, transcription activator-like effector nucleases, and meganucleases. Future improvements in CRISPR/Cas9 gene editing may be achieved by increasing the efficiency of homology-directed repair. Here, we describe principles of genetic engineering and detail: (1) how common elements of current technologies include the need for a chromosome break to occur, (2) the use of specific and sensitive genotyping assays to detect altered genomes, and (3) delivery modalities that impact characterization of gene modifications. In summary, while some principles of genetic engineering remain steadfast, others change as technologies are ever-evolving and continue to revolutionize research in many fields.

## 1. Introduction

Since the identification of DNA as the unit of heredity and the basis for the central dogma of molecular biology [1] that DNA makes RNA and RNA makes proteins, scientists have pursued experiments and methods to understand how DNA controls heredity. With the discovery of molecular biology tools such as restriction enzymes, DNA sequencing, and DNA cloning, scientists quickly turned to experiments to change chromosomal DNA in cells and animals. In that regard, initial experiments that involved the co-incubation of viral DNA with cultured cell lines progressed to the use of selectable markers in plasmids. Delivery methods for random DNA integration have progressed from transfection by physical co-incubation of DNA with cultured cells, to electroporation and microinjection of cultured cells [2,3,4]. Moreover, the use of viruses to deliver DNA to cultured cells has progressed in tandem with physical methods of supplying DNA to cells [5,6,7]. Homologous recombination in animal cells [8] was rapidly exploited by the mouse genetics research community for the production of gene-modified mouse ES cells, and thus gene-modified whole animals [9,10].

This impetus to understand gene function in intact animals was ultimately manifested in the international knockout mouse project, the purpose of which was to knock out every gene in the mouse genome, such that researchers could choose to make knockout mouse models from a library of gene-targeted knockout ES cells [11,12,13]. Thousands of mouse models have resulted from that effort and have been used to better understand gene function and the bases of human genetic diseases [14]. This project required high-throughput pipelines for the construction of vectors, including bacterial artificial chromosome (BAC) recombineering technology [13,15,16,17]. BACs contain long segments of cloned genomic DNA. For example, the C57BL/6J mouse BAC library, RPCI-23, has an average insert size of 197 kb of genomic DNA per clone [18]. Because of their size, BACs often carry all of the genetic regulatory elements to faithfully recapitulate the expression of genes contained in them, and thus can be used to generate BAC transgenic mice [19,20]. Recombineering can be used to insert reporters in BACs that are then used to generate transgenic mice to accurately label cells and tissues according to the genes in the BACs [21,22,23,24,25,26]. A panoply of approaches to genetic engineering are available for researchers to manipulate the genome. ES cell and BAC transgene engineering have given way to directly editing genes in zygotes, consequently avoiding the need for ES cell or BAC intermediates on the way to an animal model.

Prior to the adaptation of Streptococcus pyogenes Cas9 protein to cause chromosome breaks, three other endonuclease systems were used: (1) rare-cutting meganucleases, (2) zinc finger nucleases (ZFNs), and (3) transcription activator-like effector (TALE) nucleases (TALENs) [27]. The I-CreI meganuclease recognizes a 22 bp DNA sequence [28,29]. Proof-of-concept experiments demonstrated that the engineered homing endonuclease I-CreI can be used to generate transgenic mice and transgenic rats [30]. I-CreI specificity can be adjusted to target specific sequences in DNA by protein engineering methodology, although this limits its widespread application to genetic engineering [31]. Subsequently, ZFN technology was developed to cause chromosome breaks [32]. A single zinc finger is made up of 30 amino acids that bind three base pairs. Thus, three zinc fingers can be combined to specifically recognize nine base pairs on one DNA strand and a triplet of zinc fingers is made to bind nine base pairs on the opposite strand. Each zinc finger is fused to the DNA-cutting domain of the FokI restriction endonuclease. Because FokI domains only cut DNA when they are present as dimers, a ZFN monomer binding to a chromosome cannot induce a DNA break [32], instead requiring ZFN heterodimers for sequence-specific chromosome breaks. It is estimated that 1 in every 500 genomic base pairs can be cleaved by ZNFs [33]. Compared with meganucleases, ZFNs are easier to construct because of publicly available resources [34]. Additionally, the value of ZFNs in mouse and rat genome engineering was demonstrated in several studies that produced knockout, knockin, and floxed (described below) animal models [35,36,37]. The development of transcription activator-like effector nucleases (TALENs) followed after ZFN technology [38]. TALENs are made up of tandem repeats of 34 amino acids. The central amino acids at positions 12 and 13, named repeat variable di-residues (NVDs), determine the base to which the repeat will bind [38]. To achieve a specific chromosomal break, 15 TALE repeats assembled and fused to the FokI endonuclease domain (TALEN monomer) are required. Thus, one TALEN monomer binds to 15 base pairs on one DNA strand, and a second TALEN monomer binds to bases on the opposite strand [38]. When the FokI endonuclease domains are brought together, a double-stranded DNA break occurs. In this way, a TALEN heterodimer can be used to cause a sequence-specific chromosome break. It has been estimated that, within the entire genome, TALENs have potential target cleavage sites every 35 bp [39]. Compared with ZFNs, TALENs are easier to construct with publicly available resources [40,41], and TALENs have been adopted for use in mouse and rat genome engineering in several laboratories that have produced knockout and knockin animal models [42,43,44,45,46].

**Figure 1:**
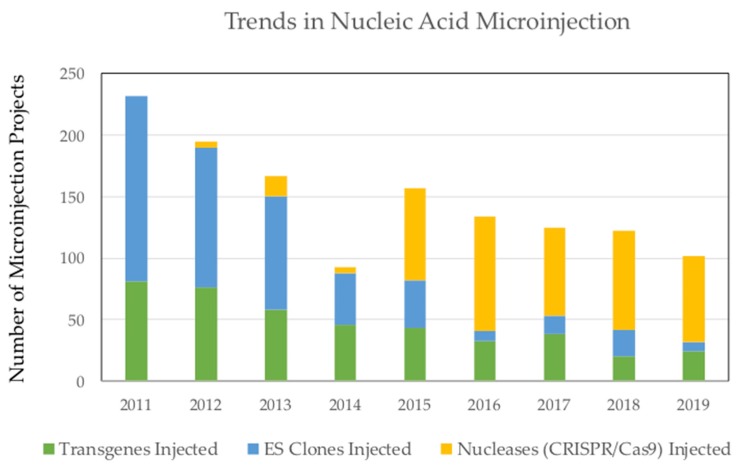
Recent trends in nucleic acid microinjection in zygotes, and embryonic stem (ES) cell microinjections into blastocysts, for the production of genetically engineered mice at the University of Michigan Transgenic Core. As shown, prior to the introduction of CRISPR/Cas9, the majority of injections were of ES cells, to produce gene-targeted mice, and DNA transgenes, to produce transgenic mice. After CRISPR/Cas9 became available, adoption was slow until 2014, when it was enthusiastically embraced, and the new technology corresponded to a reduced demand for ES cell and DNA microinjections.

The efficiencies of producing specific double-strand chromosome breaks, using prior technologies such as meganucleases, ZFNs, and TALENs [28,32,38], were surpassed when CRISPR/Cas9 technology was shown to be effective in mammalian cells [47,48,49]. The essential feature that all of these technologies have in common is the production of a chromosome break at a specific location to facilitate genetic modifications [50]. In particular, the discovery of bacterial CRISPR-mediated adaptive immunity, and its application to genetic modification of human and mouse cells in 2013 [47,48,49], was a watershed event to modern science. Moreover, the introduction of CRISPR/Cas9 methodology has revolutionized transgenic mouse generation. This paradigm shift can be seen by changes in demand for nucleic acid microinjections into zygotes, and ES cell microinjections into blastocysts at the University of Michigan Transgenic Core (Figure 1). While previously established principles of genetic engineering using mouse ES cell technology [51,52,53] remain applicable, CRISPR/Cas9 methodologies have made it much easier to produce genetically engineered model organisms in mice, rats, and other species [54,55]. Herein, we discuss principles in genetic engineering for the design and characterization of targeted alleles in mouse and rat zygotes, or in cultured cell lines, for the production of animal and cell culture models for biomedical research. 

## 2. Principles of Genetic Engineering

### 2.1. Types of Genetic Modifications

There are many types of genetic modifications that can be made to the genome. The ability to specifically target locations in the genome has expanded our ability to make changes that include knockouts (DNA sequence deletions), knockins (DNA sequence insertions), and replacements (replacement of DNA sequences with exogenous sequences). Deletions in the genome can be used to knockout gene expression [56,57]. Short deletions in the genome can be used to remove regulatory elements that knockout gene expression [58], activate gene expression [59], or change protein structure/function by changing coding sequences [60].

Insertion of new genomic information can be used to knock in a variety of genetic elements. Knockins are also powerful approaches for modifying genes. Just as genomic deletions can be used to change gene function, knockins can be used to block gene function by inserting fluorescent reporter genes such as eGFP or mCherry, in such a way as to knock out the gene at the insertion point [61,62]. It is also possible to knock in fluorescent protein reporter genes, without knocking out the targeted gene [63,64]. Just as fluorescent proteins can be used to label proteins and cells, short knockins of epitope tags in proteins can be used to label proteins for detection with antibodies [64,65].

Replacement of DNA sequences in the genome can be used to achieve two purposes at the same time, such as blocking gene function, while activating the function of a new gene such as the lacZ reporter [66]. Large-scale sequence replacements are possible with mouse ES cell technology, such as the replacement of the mouse immunoglobulin locus with the human immunoglobulin locus to produce a “humanized” mouse [67]. Furthermore, very small replacements of single nucleotides can be used to model point mutations that are suspected of causing human disease [68,69,70].

A special type of DNA sequence replacement is the conditional allele. Conditional alleles permit normal gene expression until the site-specific Cre recombinase removes a loxP-flanked critical exon to produce a “floxed” (flanked by loxP) exon. Cre recombinase recognizes 34 bp loxP (locus of recombination) elements, and catalyzes recombination between the two loxP sites [71,72]. Therefore, deletion of the critical exon causes a premature termination codon to occur in the mRNA transcript, triggering its nonsense-mediated decay and failure to make a protein [13,73]. Engineering conditional alleles was the approach used by the international knockout mouse project [13]. Mice with cell- and tissue-specific Cre recombinase expression are an important resource for the research community [74].

Other site-specific recombinases, such as FLP, Dre, and Vika, that work on the same principle have also been applied to mouse models [75,76,77,78,79,80]. Recombinase knockins can be designed to knock out the endogenous gene or preserve its function [81,82]. A variation in the conditional allele is the inducible allele, which is silent until its expression is activated by Cre recombinase [79]. For example, reporter models can activate the expression of a fluorescent protein [83], change fluorescent reporter protein colors from red to green [84], or use a combinatorial approach to produce up to 90 fluorescent colors [85]. Another type of inducible allele is the FLEX allele. FLEX genes are Cre-dependent gene switches based on the use of heterotypic loxP sites [86]. In one application that combined Cre and FLP recombinases, it was demonstrated that a gene inactivated in ES cells by a gene trap could be switched back on and then switched off again [87]. In another application of heterotypic loxP sites in mouse ES cells, it was demonstrated that genes could be made conditional by inversion (COIN) [88]. This application has been used to produce mice with conditional genes for point mutations [89] and has been applied to produce conditional single exon genes that lack critical exons by definition [90].

### 2.2. Genetic Engineering with CRISPR/Cas9 

The central principle of gene targeting with CRISPR/Cas9, or other directed DNA endonucleases, is that a double-strand DNA break is generated in the cell of interest. Following a chromosomal break, the principal outcomes of interest are nonhomologous end joining (NHEJ) repair [91] or homology-directed repair (HDR) [92]. When the break is directed to a coding exon in a gene, the outcome of NHEJ is usually a small insertion or deletion of DNA sequence at the break (indel), causing frame shifts in mRNA transcripts that lead to premature termination codons, causing nonsense-mediated mRNA decay and loss of protein expression [73]. The HDR pathway copies a template during DNA repair, and thus the insertion of modified genetic sequences in the form of a DNA donor. This DNA donor can introduce new information into the genome flanked by homology arms on either side of the chromosome break. Typical applications of HDR include the use of genetic engineering to abrogate gene expression (gene knockouts), to modify amino acid codons (i.e.; point mutations), to replace genes with new genes (e.g.; knockins of fluorescent reporters, Cre recombinase, cDNA coding sequences), to produce conditional genes (floxed genes that are normally expressed until they are inactivated by Cre recombinase), to produce Cre-inducible genes (genes that are only expressed after Cre recombinase activates them), and to delete DNA from chromosomes (e.g.; delete regulatory elements that control gene expression, delete entire genes, or delete up to a megabase of chromosome segments). The simplest of these modifications is abrogation of gene expression. Multifunctional alleles, such as FLEX alleles, require the cloning or synthesis of multi-element plasmid DNA donors for HDR.

The processes of CRISPR/Cas9-mediated modifications of genes (gene editing) to produce a new cell line or animal model have in common a series of steps to achieve the final product. First, a gene of interest is identified and the final desired allele is specified. The next step is to identify single guide RNA(s) (gRNAs) that will be used to target a chromosomal break in one or more places. There are numerous online websites that can be used for this purpose [93]. One of the most up-to-date and versatile sites is CRISPOR (http://crispor.tefor.net) [94]. Interestingly, the authors provide evidence that the predictive powers of algorithms vary depending on whether they were based on the analysis of gRNAs delivered as RNA molecules, versus gRNAs delivered as U6-transcribed DNA molecules [94]. In any event, the selection of a gRNA target (20 nucleotides), adjacent to a protospacer-adjacent motif (PAM; NGG motif), should not be done without the aid of a computer algorithm that minimizes the possibility of off-target hits. After a gRNA target is identified, a decision is made to obtain gRNAs. While it is possible to produce in vitro-transcribed gRNAs, this may be inadvisable in so much as in vitro-transcribed RNAs can trigger innate immune responses and cause cytotoxicity in cells [95]. Chemically synthesized gRNAs using phosphorothioate modifications that improve gRNA stability may be preferable alternatives to in vitro-transcribed molecules [96,97]. With a gRNA in hand, a Cas9 protein is then selected. There are numerous forms of Cas9 that can be used for different purposes [98]. For practical purposes, we limit our discussion to Cas9 varieties that are on the market. A number of commercial entities sell wild-type Cas9 protein. When wild type Cas9 is used to target the genome with nonspecific guides, the frequency of off-target genomic hits, besides the desired Cas9 target, is very likely to increase [94,99]. Alternatives to the wild-type protein include enhanced specificity Cas9 from Sigma-Aldrich [100], and high-fidelity Cas9 from Integrated DNA Technologies [101]. In addition, there are other versions such as HF1 Cas9 [102], hyperaccurate Cas9 [103], and evolved Cas9 [104], all available in plasmid format from Addgene.org. As may be inferred from the names of these engineered Cas9 versions, they are designed to be more specific than wild type Cas9. Once the gRNAs and Cas9 protein are on hand, then it is a “simple” matter to combine them and deliver them to the target cell to produce a chromosome break and achieve a gene knockout by introducing premature termination codons or DNA sequence deletion of regulatory regions or entire genes.

### 2.3. Locus-Specific Genetic Engineering Vectors in Mouse and Rat Zygotes

The most challenging type of genetic engineering is the insertion (i.e.; knockin) of a long coding sequence to express a fluorescent reporter protein, Cre recombinase, or conditional allele (floxed gene). In addition to these genetic modifications, numerous other types of specialized reporters can be introduced, each designed to achieve a different purpose. There is great interest in achieving rapid and efficient gene insertions of reporters in animal models with CRISPR/Cas9 technology. It is generally recognized that, the longer the insertion, the less efficient it is to produce a knockin animal. Additional challenges are allele-specific differences that affect efficiency. For example, it is fairly efficient to produce knockins into the genomic ROSA26 locus in mice, while other loci are targeted less efficiently, and thus refractory to knockins. This accessibility to CRISPR/Cas9 complexes mirrors observations in mouse ES cell gene targeting technology, in which it was reported that some genes are not as efficiently targeted as others [105].

When the purpose of the experiment is to specifically modify the DNA sequence by changing amino acid codons, or introducing new genetic information, then a DNA donor must be delivered to the cells with Cas9 reagents. After the selected gRNAs and Cas9 proteins are demonstrated to produce the desired chromosome break, the DNA donor is designed and procured. The donor should be designed to insert into the genome such that it will not be cleaved by Cas9, usually by mutating the PAM site. The DNA donor may take the form of short oligonucleotides (<200 nt) [106,107], long single-stranded DNA molecules (>200 nt) [108], or double-stranded linear or circular DNA molecules of varying lengths [109,110].

DNA donor design principles should include the following: (1) nucleotide changes that prevent CRISPR/Cas9 cleavage of the chromosome, after introduction of the DNA donor; (2) insertion of restriction enzyme sites unique to the donor, to simplify downstream genotyping; (3) insertions of reporters or coding sequences, at least 1.5 kb in length, that can be introduced as long single-stranded DNA templates with short 100 base pair arms of homology [111], or as circular double-stranded DNA plasmids with longer (1.5 or 2 kb) arms of homology [63,110]; and (4) insertions of longer coding sequences, such as Cas9, that use circular double-stranded DNA donors with longer arms of homology [63,112]. It is also possible to use linear DNA fragments as donors [63,110,113], although random integration of linear DNA molecules is much higher than those of circular donors, thus requiring careful quality control.

The establishment of genetically modified mouse and rat models can be divided into three phases, after potential founder animals are born from CRISPR/Cas9-treated zygotes. In the first phase, animals with genetic modifications are identified. The first phase requires a sensitive and specific genotyping assay to identify cells or animals harboring the desired knockin. Genotyping potential founder mice for knockins typically begins with a PCR assay using a primer that recognizes the exogenous DNA sequence and a primer in genomic DNA outside of the homology arm in the targeting vector. Accordingly, PCR assays are designed to specifically detect the upstream and downstream junctions of the inserted DNA in genomic DNA. Subsequent assays may be used to confirm that the entire exogenous sequence is intact. Conditional genes represent a special case of insertion, as PCR assays designed to detect correct insertion of loxP-flanked exons will also detect genomic DNA [108]. In the second phase, founders are mated and G1 pups are identified that inherited the desired mutation [114]. In the third phase, it is essential to sequence additional genomic regions upstream and downstream of the inserted targeting vector DNA, because Cas9 is very efficient at inducing chromosomal breaks, but has no repair function. Thus, it is not unusual to identify deletions/insertions that flank the immediate vicinity of the Cas9 cut site or inserted targeting vector DNA sequences [115,116]. If such deletions affect nearby exons, gene expression can be disrupted, and confounding phenotypes may arise.

For gene knockouts, PCR amplicons from primers that span the chromosome break site are analyzed by DNA sequencing. Any animals that are wild-type at the allele are not further characterized or used, so as to prevent any off-target hits from entering the animal colony or confounding phenotypes. Animals that show disrupted DNA sequences at the Cas9 cut site are mated with wild-type animals for the transmission of mutant alleles that produce premature termination codons, for gene knockout models [57,73]. As founders from Cas9-treated zygotes are genetic mosaics [55,115], it is essential to mate them to wild-type breeding partners, such that obligate heterozygotes are produced. In the heterozygotes, the wild-type sequence and the mutant sequence can be precisely identified by techniques such as TOPO TA cloning (Invitrogen, CA, USA) or next-generation sequencing (NGS) methods [117,118,119,120]. Animals carrying a defined indel, with the desired properties, are then used to establish lines for phenotyping. The identical approach is used when short DNA sequences are deleted by two guide RNAs [58]. Intercrossing mosaic founders will produce offspring carrying two different mutations with different effects on gene expression. These animals are not suitable for line establishment.

### 2.4. Gene Editing in Immortalized Cell Lines 

CRISPR/Cas9 gene editing in immortalized cell lines presents a set of challenges unique from those used in the generation of transgenic animals. Cell lines encompass a wide range of characteristics, resulting in each line being handled differently. Some of these characteristics include phenotype heterogeneity, aberrant chromosome ploidy, varying growth rates, DNA damage response efficiency, transfection efficiency, and clonability. While the principles of CRISPR/Cas9 experimental design, as stated above, remain the same, three major considerations must be taken into account when using cell lines: (1) copy number variation, or the number of alleles of the gene of interest; (2) transfection efficiency of the cell line; and (3) clonal isolation of the modified cell line. In cell lines, all alleles need to be modified in the generation of a null phenotype, or in the creation of a homozygous genotype. Unlike transgenic animals, where single allele gene edits can be bred to homozygosity, CRISPR/Cas9-edited cells must be screened for homozygous gene edits. Copy number variations within the cell line can decrease the efficiency and add labor and time (i.e.; editing 3 or 4 copies versus editing 1 or 2). Furthermore, an aberrant number of chromosomes, deletions, duplications, pseudogenes, and repetitive regions complicate genetic backgrounds for PCR analysis of the CRISPR edits. To help with some of these issues, one common approach is to use NGS on all the clonal isolates for a complete understanding of copy number variations for each clonal cell line generated, and the exact sequence for each allele.

As all cell types are not the same, different CRISPR/Cas9 delivery techniques may need to be tested to identify which method works best. One approach is to use viruses or transposons to deliver CRISPR/Cas9 reagents (detailed below). However, the viruses and transposons themselves will integrate into the genome, as well as allowing long-term expression of CRISPR/Cas9 in the cell. This prolonged expression of gRNAs and Cas9 protein may lead to off-target effects. Moreover, transfection and electroporation can have varying efficiencies, depending on the cell lines and the form of CRISPR/Cas9 reagents (e.g.; DNA plasmids or ribonucleoprotein particles (RNPs)).

Following delivery, clonal isolation is required to identify the edited cell line, and at times, can result in the isolation of a cell phenotype different than that expected, arising from events apart from the desired gene edit. While flow cytometry can aid in isolating individual cells, specific flow conditions, such as pressure, may require adjustment to ensure cell viability. Furthermore, one clonal isolate from a cell line may possess a different number of alleles for the targeted gene than another clonal isolate. Additionally, not all cell lines will grow from a single cell, thus complicating isolation. Growth conditions and cell viability can also change when isolating single cells.

Despite these challenges, new advances in CRISPR technology can likely alleviate some of these difficulties when editing cell lines. For example, fluorescently tagged Cas9 and RNAs help to isolate only transfected cells, which helps to eliminate time wasted on screening untransfected cells. Cas9-variants that harbor mutations that only create single-strand nicks (Cas9-nickases) complexed with two different, but proximal gRNAs can increase HDR-mediated knockin [48,121]. Similarly, fusing Cas9 with base-editing enzymes can also increase the efficiency of editing, without causing double-strand breaks [121].

### 2.5. Viruses and Transposons as Genetic Engineering Vectors

Viral and transposon vectors have been engineered to be safe, efficient delivery systems of exogenous genetic material into cells. The natural lifecycle of some viruses and transposons includes the stable integration into the host genome. In the field of genome engineering, these vectors can be used to modify the genome in a non-directed fashion, by inserting cassettes expressing any cDNA, shRNA, miRNA, or any non-coding RNA. The most widely used vectors capable of integrating ectopic genetic material into cells are retroviruses, lentiviruses, and adeno-associated virus (AAV). These viruses are flanked by terminal repeats that mark the boundaries of the integration. In engineering these viruses into recombinant vector systems, all the viral genes are removed from the flanking terminal repeats and supplied in trans for the recombinant virus to be packaged. These “gutted”, nonreplicable viral vectors allow for the packaging, delivery, integration, and expression of cDNAs of interest, shRNAs, and CRISPR/Cas9, without viral replication in various biological targets.

Similar to recombinant viruses, transposon vectors are also “gutted”, separating the transposase from the terminal repeat-flanked genetic material to be inserted into the genome. DNA transposons are mobile elements (“jumping genes”) that integrate into the host genome through a cut-and-paste mechanism [122]. Transposons, much like viral vectors, are flanked by repeats that mark the region to be transposed [123]. The enzyme transposase binds the flanking DNA repeats and mediates the excision and integration into the genome. Unlike viral vectors, transposons are not packaged into viral particles, but form a DNA-protein complex that stays in the host cell. Thus, the transgene to be integrated can be much larger than the packaging limits of some viruses.

Two transposons, Sleeping Beauty (SB) and piggybac (PB), have been engineered and optimized for high activity for generating transgenic mammalian cell lines [124,125,126]. Sleeping Beauty is a transposable element resurrected from fish genomes. The SB system has been used to generate transgenic HeLa cell lines, T-cells expressing chimeric antigen receptors that recognize tumor-specific antigens, and transgenic primary human stem cells [127,128,129]. The insect-derived PB system also has been used to generate transgenic cell lines [126,130,131]. The PB system was used to generate induced pluripotent stem cells (iPSCs) from mouse embryonic fibroblasts, by linking four or five cDNAs of the reprogramming (Yamanaka) factors [132] with intervening peptide self-cleavage (P2A) sites, thus delivering all of the factors in one vector [130]. Furthermore, once reprogrammed, the transgene may be removed by another round of PB transposase activity, leaving no genetic trace of integration or excision (i.e.; transgene-free iPSCs). Following PB transposase activity, epigenetic differences remaining at the endogenous promoters of the reprogramming factor genes result in sustained expression and pluripotency, despite transgene removal.

Aside from transgene insertion, Sleeping Beauty (SB) and piggyback (PB) have both been engineered to deliver CRISPR/Cas9 reagents into cells [133,134,135]. Similar to lentivirus, the stable integration of CRISPR/Cas9 by transposons could increase the efficacy of targeting and modifying multiple alleles. SB and PB have been used to deliver multiple gRNAs to target multiple genes (instead of just one), aiding in high-throughput screening. Furthermore, owing to the nature of PB excision stated above, the integrated CRISPR/Cas9 can be removed once a clonal cell line is established, to limit off-target effects. However, engineered transposons must be transfected into cells. As stated above, efficiencies vary between different cell lines and transfection methods. One potential solution to overcome this challenge is to merge technologies. For example, instead of transfecting cells with a plasmid harboring a gRNA flanked by SB terminal repeats (SB-CRISPR), the SB-CRISPR may be flanked by recombinant AAV (rAAV) terminal repeats (AAV-SB-CRISPR), allowing for packaging into rAAV. To that end, rAAV-SB-CRISPR has been used to infect primary murine T-cells, and deliver the SB-CRISPR construct [136].

### 2.6. Genetic Engineering Using Retroviruses 

Retroviruses are RNA viruses that replicate through a DNA intermediate [137]. They belong to a large family of viruses including both onco-retroviruses, such as the Moloney murine leukemia virus (MMLV) (simply referred to as retrovirus), and lentiviruses, including human immunodeficiency virus (HIV). In all retroviruses, the RNA genome is flanked on both sides by long terminal repeats (LTRs); packaged with viral reverse transcriptase, integrase, and protease, surrounded by a protein capsid; and then enveloped into a lipid-based particle [138]. Envelope proteins interact with specific host cell surface receptors to mediate entry into host cells through membrane fusion. Then, the RNA genome is reverse-transcribed by the associated viral reverse transcriptase. The proviral DNA is then transported into the nucleus, along with viral integrase, resulting in integration into the host cell genome [139]. By contrast, the retroviral MMLV pre-integration complex is incapable of crossing the nuclear membrane, thus requiring the cell to undergo mitosis to gain access to chromatin [139], while lentiviral pre-integration complexes can cross nuclear membrane pores, allowing genome integration in both dividing and non-dividing cells.

Large-scale assessments of genomic material composition have uncovered features associated with retroviral insertion into mammalian genomes [140]. Although determination of integration target sites remains ill-defined, it does depend on both cellular and viral factors. For retroviruses such as MMLV, integration is preferentially targeted to promoter and regulatory regions [140,141,142]. Such preferences can be genotoxic owing to insertional activation of proto-oncogenes in patients undergoing gene therapy treatments for X-linked severe combined immunodeficiency [143,144], Wiskott–Aldrich syndrome [143], and chronic granulomatous disease [145]. Likewise, retroviral integration can generate chimeric and read-through transcripts driven by strong retroviral LTR promoters, post-transcriptional deregulation of endogenous gene expression by introducing retroviral splice sites (leading to aberrant splicing), and retroviral polyadenylation signals that lead to premature termination of endogenous transcripts [142,146,147].

Unlike retroviruses, lentiviruses prefer to integrate into transcribed portions of expressed genes in gene-rich regions, distanced from promoters and regulatory elements [140,142,148]. The cellular protein LEDGF/p75 aids in the target site selection by binding directly to both the active gene and the viral integrase within the HIV pre-integration complex [149]. Although the propensity of lentivirus to integrate into the body of expressed genes should increase the incidence of post-transcriptional deregulation, deletion of promoter elements from the lentiviral LTR (self-inactivating (SIN) vectors) has been reported to decrease transcriptional termination, but increase the generation of chimeric transcripts [149]. Overall, it appears that lentiviral SIN vectors are less likely to cause tumors than retroviral vectors with an active LTR promoter [148,150,151,152].

The 7.5–10 kb packaging limit of lentiviruses can accommodate the packaging, delivery, and stable integration of Cas9 cDNA, gRNAs, or Cas9 and gRNAs (all-in-one) to cells [153,154]. Often, a selectable marker, such as drug resistance, can also be included to isolate transduced cells. The high transduction efficiency of lentivirus can result in an abundance of CRISPR/Cas9-expressing cells to screen, compared with more traditional transfection methods. Stable and prolonged expression of CRISPR/Cas9 can facilitate targeting of multiple alleles of the gene of interest, resulting in more cells harboring homozygous gene modifications. Conversely, stable integration of CRISPR/Cas9 increases potential off-target effects. Moreover, lentiviral integration itself is a factor that may confound cellular phenotypes and should be considered when characterizing CRISPR-edited cell lines.

### 2.7. Gene Targeting Using Adeno-Associated Virus 

Adeno-associated virus (AAV) is a human parvovirus with a single-stranded DNA genome of 4.7 kb, which was originally identified as a contaminant of adenoviral preparations [155]. The genome is flanked on both sides by inverted terminal repeats (ITR) and contains two genes, *rep* and *cap* [156,157]. Different capsid proteins confer serotype and tissue-specific targeting of distinct AAVs, in vivo. AAV cannot replicate on its own, and requires a helper virus, such as adenovirus or herpes simplex virus (HSV), to provide essential proteins in trans. AAV is the only known virus to integrate into the human genome in a site-specific manner at the AAVS1 site on chromosome 19q13.3-qter [158,159,160]. Although the precise mechanism is not well understood, the Rep protein functions to tether the virus to the host genome through direct binding of the AAV ITR and the AAVS1 site [158,160,161]. In the recombinant AAV (rAAV) vector system, the *rep* and *cap* genes are removed from the packaged virus, resulting in the loss of site-specific integration into the AAVS1 site. Despite removal of Rep, it has been shown that rAAV can still integrate, albeit randomly, into the host genome, via nonhomologous recombination, at low frequencies [162,163,164]. Furthermore, numerous clinical trials, to date, have shown that rAAV integration is safe and has no genotoxicity [165,166,167]. However, this “safety” is controversial, owing to preclinical studies suggesting genotoxicity in mouse models [168,169,170,171]. More studies are needed to understand the cellular impact of rAAV integration.

rAAVs have been used to deliver one or two CRISPR guide RNAs (gRNAs), in cells and model animals, by taking advantage of different rAAV serotypes to target specific cells or tissue types. Owing to the packaging capacity of rAAV, SpCas9 must be delivered as a separate virus, unlike lentivirus, which can be delivered as an “all-in-one” CRISPR/Cas9 vector. However, alternate, smaller Cas9s can be packaged into rAAVs [172]. Furthermore, rAAVs can be used to deliver repair templates or single-stranded donor oligonucleotides (ssODNs) for homology-directed repair (HDR), relying on the single-stranded nature of the AAV genome [173,174]. It has also been observed that rAAVs can integrate into the genome at CRISPR/Cas9-induced breaks in various cultured mouse tissue types, including neurons and muscle [175]. This observation goes against the notion of rAAVs integrating only at the AAVS1 locus, and should be considered when analyzing and characterizing rAAV-mediated CRISPR-edited cells.

## 3. Conclusions

There are many approaches to inserting new genetic information into chromosomes in cells and animals. At this time, the most appealing method is single copy gene insertion at a defined locus. This approach has numerous advantages, with respect to reproducible transgene expression. Random insertion transgenesis has been effectively used to probe gene function in mouse models [176]. It is generally accepted that this requires a spontaneous chromosome break [176]. Recent NGS data suggest that the repair mechanism resembles chromothripsis [118,177]. In addition to unintended gene disruptions owing to chromosome damage, the random insertion of transgenes exposes them to “position effects” in which their expression is controlled by neighboring genes [118,178]. Ideally, the insertion of reporter cDNAs in the genome results in single copy transgene insertions in defined loci in such a way that endogenous genes are not disrupted, and reporters are placed under the control of specific endogenous promoters [179]. The application of CRISPR/Cas9 technology to address this problem shows it can be used to achieve these goals [63,82,180]. The development of CRISPR/Cas9 base editing technology shows that it is possible to make single-nucleotide changes in the genome [181,182,183,184]. Base editors have the advantage that double-strand chromosome breaks are not produced, thus lessening the chances of undesirable mutations in the genome. A novel approach to small insertions in the genome by the use of a RNA donor sequence fused to the sgRNA in combination with a reverse transcriptase fused to dead Cas9 also avoids the need to produce double-strand breaks on chromosomes. This approach is referred to as “prime editing” [185]. CRISPR technology that avoids chromosome breaks, while making changes to the genome, is extremely important in clinical applications where unintended changes can adversely affect patients. These advanced versions of CRISPR technology will be important for future research.

The desire to apply CRISPR/Cas9 for the targeted insertion of transgenes is reflected in the profusion of methods directed towards this purpose [63,108,110,112,186,187]. Each method was successfully used to engineer mouse and rat genomes (Table 1). Each method was shown to be more cost-effective and rapid than the application of mouse or rat ES cell technology. For the practitioner of the art, the question remains: which method is most efficient? That is to say, which method minimizes the number of animals needed for zygote production and maximizes the number of gene-targeted founders? One approach to this question is to compare the transgenic efficiency of each method [188]. The results in Table 1 show that the highest efficiency experiments were obtained when long single-stranded DNA donors and Cas9 ribonucleoproteins were used to produce genetically engineered mice. All methods are very effective compared with traditional methods of gene targeting in zygotes. Perhaps future avenues to even more efficient gene targeting lie in the application of small molecule activators for HDR [189,190,191].

## Figures and Tables

**Table 1:** Analysis of targeting vector knockin by CRISPR/Cas9 in mouse and rat zygotes.

Targeted Gene	Purpose ^1^	Cas9 Format ^2^	DNA Donor Format ^3^	Efficiency ^4^	Reference
Pitx1	Conditional	RNP	ssDNA	5.3	[108]
Ambra1	Conditional	RNP	ssDNA	9.5	[108]
Col12a1	Conditional	RNP	ssDNA	3.8	[108]
Ubr5	Conditional	RNP	ssDNA	12.5	[108]
Syt1	Conditional	RNP	ssDNA	2.2	[108]
Syt9	Conditional	RNP	ssDNA	2.4	[108]
PPP2r2a	Conditional	RNP	ssDNA	9.1	[108]
Fgf8	Reporter	RNP	ssDNA	7.7	[108]
Slc26a5	Reporter	RNP	ssDNA	4.5	[108]
Mafb	Reporter	RNP	ssDNA	3.8	[108]
Otoa	Reporter	RNP	ssDNA	5.6	[108]
Mmp9	Reporter	RNP	ssDNA	16.0	[108]
Mmp13	Reporter	RNP	ssDNA	7.7	[108]
Sox2	Reporter	Cas9 mRNA	dsDNA	2.0	[63]
Nanog	Reporter	Cas9-mSA	BioPCR	2.7	[63]
Gata6	Reporter	Cas9 mRNA	dsDNA	2.0	[63]
Gata6	Reporter	Cas9-mSA	BioPCR	5.0	[63]
Cdk9	Reporter	Cas9 mRNA	dsDNA	4.0	[63]
ROSA26	Reporter	Cas9 mRNA	dsDNA	1.3	[63]
Cdx2	Reporter	Cas9 mRNA	HMEJ	5.9	[110]
Cdx2	Reporter	Cas9 mRNA	Tild	1.9	[110]
Dbh	Reporter	Cas9 mRNA	Tild	3.6	[110]
Sp8	Reporter	Cas9 mRNA	HMEJ	3.2	[110]
Sp8	Reporter	Cas9 mRNA	Tild	2.0	[110]
Tdtomato	Reporter	Cas9 mRNA	Tild	3.5	[110]
Nr3c2	Conditional	Cas9 mRNA	Tild	4.8	[110]
Lhx6	Conditional	Cas9 mRNA	Tild	6.3	[110]
Serpina3	Conditional	Cas9 mRNA	ssDNA	3.5	[186]
Tyr	Conditional	Cas9 mRNA	ssDNA	2.0	[186]
mKIAA1322	Conditional	Cas9 mRNA	ssDNA	3.0	[186]
Serpina3n	Conditional	Cas9 mRNA	ssDNA	1.3	[186]
Mct4	Conditional	Cas9 mRNA	ssDNA	1.5	[186]
Rat Vapb	Conditional	Cas9 mRNA	ssDNA	3.9	[186]
ROSA26	Reporter	RNP	AAV	1.2	[187]
ROSA26	Reporter	RNP	AAV	4.8	[187]
Rat ROSA26	Reporter	RNP	AAV	4.2	[187]
Rat ROSA26	Reporter	RNP	AAV	5.4	[187]
ROSA26	Reporter	Cas9 mRNA	dsDNA	3.4	[112]
ROSA26	Reporter	Cas9 mRNA	dsDNA	2.1	[112]

^1^ Conditional: A critical exon was flanked by loxP sites, so as to produce a Cre-dependent knockout allele. Reporter: an exogenous coding sequence, such as for a fluorescent protein, was inserted. ^2^ RNP: ribonucleoprotein; Cas9 protein was complexed with guide RNA. Cas9 mRNA: in vitro transcribed mRNA from a plasmid containing Cas9 mixed with guide RNA. Cas9-mSa: in vitro transcribed mRNA from a plasmid containing Cas9 fused to monomeric streptavidin. ^3^ ssDNA: single-stranded DNA repair template. BioPCR: PCR was used to prepare biotinylated PCR amplicons. dsDNA: circular double-stranded DNA repair template. HMEJ: homology-mediated end joining; circular double-stranded DNA repair template incorporating sgRNA targets that flank homology arms. Tild: linear double-stranded DNA repair template. AAV: an adeno-associated vector donor was cultured with zygotes loaded with Cas9 RNP, by electroporation. ^4^ Efficiency, as calculated as the number of genetically engineered mice or rats produced per 100 zygotes treated with CRISPR/Cas9 reagents and transferred to pseudopregnant females.
